# Wider Angle Egg Turning during Incubation Enhances Yolk Utilization and Promotes Goose Embryo Development

**DOI:** 10.3390/ani11092485

**Published:** 2021-08-24

**Authors:** Binbin Guo, Leyan Yan, Mingming Lei, Zichun Dai, Zhendan Shi

**Affiliations:** Laboratory of Animal Improvement and Reproduction, Key Laboratory of Protected Agriculture Engineering in the Middle and Lower Reaches of Yangtze River, Ministry of Agriculture and Rural Affairs Institute of Animal Science, Jiangsu Academy of Agricultural Sciences, Nanjing 210014, China; guobinbin0801@163.com (B.G.); yanleyan198469@126.com (L.Y.); 20140036@jaas.ac.cn (M.L.); 20210064@jaas.ac.cn (Z.D.)

**Keywords:** incubation, egg turning angle, yolk utilization, embryo development, goose

## Abstract

**Simple Summary:**

The yolk of poultry eggs is the primary source of energy for embryonic development and the only source of lipids for embryonic tissue growth. In our previous studies on improving goose egg hatchability, we demonstrated that a wider 70° egg turning angle significantly increased hatchability and promoted embryonic growth as compared to the traditional 45° or 50° angles. However, the yolk utilization and the associated molecular mechanism, along with improved goose embryonic development, are not clear. In this research, we found that wider-angle egg turning during incubation upregulated the expression of genes related to lipolysis and fat digestion enzymes, as well as genes related to lipid transportation. The upregulation of these genes facilitates the efficient utilization of lipids that are stored in the yolk. We suggest that a wider egg turning angle, 70°, should be used in goose egg incubation to improve hatching performance and gosling quality.

**Abstract:**

We aimed to investigate how wide-angle turning of eggs during incubation affected yolk utilization and the associated molecular mechanism, along with improved goose embryonic development. In total, 1152 eggs (mean weight: 143.33 ± 5.43 g) were divided equally and incubated in two commercial incubators with tray turning angles adjusted differently, to either 50° or 70°. Following incubation under the standard temperature and humidity level, turning eggs by 70° increased embryonic days 22 (E22), embryo mass, gosling weight at hatching, and egg hatchability, but reduced E22 yolk mass compared with those after turning eggs by 50°. Lipidomic analyses of the yolk revealed that egg turning at 70° reduced the concentrations of 17 of 1132 detected total lipids, including diglycerides, triglycerides, and phospholipids. Furthermore, the 70° egg turning upregulated the expression of genes related to lipolysis and fat digestion enzymes, such as lipase, cathepsin B, and prosaposin, as well as apolipoprotein B, apolipoprotein A4, very low-density lipoprotein receptor, low-density lipoprotein receptor-related protein 2, and thrombospondin receptor, which are genes involved in lipid transportation. Thus, a 70° egg turning angle during incubation enhances yolk utilization through the upregulation of lipolysis and fat digestion-related gene expression, thereby promoting embryonic development and improving egg hatchability and gosling quality.

## 1. Introduction

Egg turning plays a key role in incubation [1] and involves important parameters, such as frequency, the axis of setting and turning, and turning angles [2,3,4]. The common chicken egg incubators operate at a turning angle of 45° [5,6,7], which is also used for incubating bigger-sized duck and goose eggs. However, the hatchability of goose eggs is usually much lower than that of chicken eggs [8]. First of all, this is related to the goose breeding environment, which can affect the quality of the eggs, including stocking density and environmental pathogen contamination [9,10]. Secondly, storage time and setting position also affect hatchability [11]. During incubation, low hatchability is partly the result of the not fully developed technology of heating and cooling goose eggs. Most importantly, egg turning during incubation can significantly influence goose egg hatchability. For example, increasing the turning angle from the traditional 45° to 70° or 75° significantly increases hatchability and improves gosling quality by enhancing embryonic growth [12,13]. Furthermore, egg turning during incubation prevents the embryonic adherence to the inner shell membrane [14], allows the embryo to adjust the spatial orientation in which it is located so as to assume the appropriate position for hatching [6], and promotes the use of oxygen by the embryo [15]. During the incubation period, the embryo satisfies all of its nutrient requirements from the egg yolk and egg white [16], which support embryonic growth and development. Especially during the second half of the incubation period and the early post-hatching period, the yolk is the primary source of energy (via fatty acid oxidation) for embryonic development and the only source of lipids for embryonic tissue growth [17,18,19]. Consequently, yolk utilization during incubation has an important effect on hatchling quality, growth performance, and health. The utilization of nutrients is enabled by an extra-embryonic membrane that surrounds the yolk, called the yolk sac membrane (YSM), which comprises a vascular system [14,20]. The capacity of the YSM to deliver nutrients affects the utilization of nutrients from the yolk [21].

In our previous studies on improving goose egg hatchability, we demonstrated that a wider 70° egg turning angle significantly increased hatchability and promoted the embryonic growth of incubated goose eggs [12,13]. Because the effects and mechanisms of wide-angle egg turning on yolk utilization are not well understood, the aim of this study was to investigate how the wide-angle turning of eggs affects yolk utilization and the associated molecular mechanism, along with improved embryonic development and hatchability. The molecular mechanisms of egg yolk lipid absorption and utilization were analyzed by non-targeted lipidomics and the expression of key genes in the YSM that are involved in lipid uptake, transport, and metabolism in the YSM. The results of this study will provide insights into enhancing yolk lipid utilization for the promotion of embryonic development and growth to improve gosling quality and hatchability during the incubation of eggs with wide turning angles.

## 2. Materials and Methods

### 2.1. Eggs and Experimental Design

A total of 1152 eggs, with a mean weight of 143.33 ± 5.43 g, were collected from a 44-week-old Yangzhou goose (male:female = 1:4), a synthetic white-feathered breed famous for its high egg-laying rate and good growth performance that is widely utilized by the Chinese goose industry. Eggs were stored for 3 days at 20 °C and 50% humidity in the small-end-down position. They were then equally divided into two treatment groups with three replicates and placed into two commercial incubators (SY-7680 incubator, Sanyuan Incubation Ltd., Bengbu, China). The turning angles of the incubators were adjusted to 50° and 70°. The eggs were incubated at 37–38 °C and a relative humidity of 60–70%. The eggs were turned once every 2 h. On days 7 and 18 of incubation, all eggs were candled, and unfertilized eggs and those with dead embryos were numbered and removed from the incubators. On day 28 of incubation, eggs were transferred into the hatcher with no further egg turning. Additionally, from days 9 to 28 of incubation, eggs were wetted and cooled with 38 °C water for 5 min twice a day.

### 2.2. Data and Tissue Collection

On embryonic days 7 and 18 (hereinafter referred to as E7 and E18, respectively), the fertilization rate and early (E0–E7) and middle (E8–E18) mortality were recorded. On E22, 10 eggs from each turning angle group were randomly selected. They were opened, and the air sac, egg yolk, and embryo were separated and weighed after the excessive fluid was dried off with a piece of absorbent paper. The yolks were aspirated with a syringe and placed in microcentrifuge tubes at −80 °C for fatty acid analysis. The YSM was separated from the yolk contents, rinsed in a 0.9% autoclaved saline solution, and placed in microcentrifuge tubes at −80 °C for mRNA analysis. On day 0 of age (referred to as the gosling out of the shell [H0]), the hatching body weight, yolk weight, hatchability of the fertilized eggs, and late mortality (E19–E30) were recorded.

### 2.3. Lipid Extraction and LC–MS Analysis of the Yolk

Lipidomic analysis was performed by the Shanghai Applied Protein Technology Co. Ltd. (Shanghai, China). Lipids were extracted with methyl tert-butyl ether (MTBE), as previously reported in another study [22]. Yolk samples (30 mg) were homogenized with 200 μL of water and 240 μL of pre-cooled methanol (Thermo Fisher Scientific, Waltham, MA, USA), after which 800 μL of MTBE was added, followed by ultrasonication for 20 min at 4 °C, and the samples were allowed to rest for 30 min at 20 °C. The solution was centrifuged at 14,000× *g* and 10 °C for 15 min, and the upper organic solvent layer was obtained and dried under nitrogen, dissolved in 200 μL of 90% isopropanol/acetonitrile, and analyzed using ultra-high-performance liquid chromatography (UHPLC) Nexera LC-30A C18 column (100 mm × 2.1 mm, 1.7 μm) at 45 °C.

Lipid metabolites were analyzed using a liquid chromatography-mass spectrometry (LC-MS) system (Thermo Scientific, Waltham, MA, USA) fitted with an electrospray ion source and a HyperSep^TM^C18 (Thermo Scientific, Waltham, MA, USA) column and precolumn, as described by Michopoulos et al. [23]. The sampling of the queue was performed for every eight samples using one of the quality controls (*n* = 4) samples to monitor and evaluate the stability and reliability of the experimental data.

The column eluent was analyzed by MS using positive and negative ionization modes. The initial lipidomic data were processed using LipidSearch software (version 4.1; Thermo Fisher Scientific Inc., Waltham, MA, USA) for peak recognition, lipid extraction (secondary appraisal), peak alignment, and quantitative processing. After normalization and integration using the Pareto scaling method, the processed data were imported into SIMPCA-P 15.1 (Umetrics, Umea, Sweden), which included principal component analysis (PCA), projections to latent structures discriminant analysis (PLS-DA), and orthogonal PLS-DA (OPLS-DA). Significant differences were identified based on a combination of statistically significant thresholds of variable influence on projection values (VIP values > 1.0) from the OPLS-DA model and two-tailed Student’s *t*-tests (*p* < 0.05). Metabolites were identified by comparing the retention indices and mass spectra data using an in-house database.

### 2.4. Reverse Transcription Quantitative Polymerase Chain Reaction (RT-qPCR)

RT-qPCR was performed to quantify the relative abundance of mRNA for various genes in the YSM samples. Total RNA was extracted from the TRIzol lysate (Invitrogen, Carlsbad, CA, USA), and RNA concentration was determined using a NanoDrop ND-1000 instrument (Thermo Fisher Scientific, Wilmington, DE, USA). cDNA (1 mg) was created from DNA-free RNA using a ReverTra Ace qPCR RT Kit, according to the manufacturer’s protocol (Toyobo, Osaka, Japan).

RT-qPCR was performed with a total volume of 20 μL (10 μL of 2× SYBR Premix ExTaq, 1 μL cDNA mix, 0.5 μL of each primer, and 8 μL of sterile distilled H_2_O). An ABI PRISM-7500 sequence detection system (Applied Biosystems, Foster City, CA, USA) was used to detect the amplified products. The primers were designed using Primer Premier 5.0 (Premier, Vancouver, Canada), and their sequences are listed in Table 1. RT-qPCR was performed using SYBR Premix Ex TaqTM (Roche, Basel, Switzerland). All RT-qPCR assays were performed in triplicate. The relative expression of target genes was determined using the 2^−ΔΔCT^ method [24] and was normalized to the expression levels of the β-actin internal housekeeping gene.

### 2.5. Statistical Analysis

Data, including the fertilization rate, relative embryo weight (embryo weight/egg weight), relative yolk weight (yolk weight/egg weight or hatching body weight), hatching body weight, mortality, and the hatchability of fertile eggs, were analyzed with Student’s *t*-test using SPSS version 18.0 (Statistical Package for the Social Sciences, SPSS Inc.; Chicago, IL, USA). The results of RT-qPCR are expressed as the means ±SEM of the eight samples for each turning angle group, which were analyzed with Student’s *t*-tests using SPSS version 18.0 (Statistical Package for the Social Sciences, SPSS Inc.; Chicago, IL, USA). Differences were considered significant at *p* < 0.05.

## 3. Results

### 3.1. Embryonic Development and Hatching Performance

The effects of wider-angle egg turning on hatching performance and relative yolk and embryo weights are shown in Table 2. There was no difference in the fertilization rate or early or middle mortality rates between the 70° and 50° turning angle groups. However, late mortality was significantly (*p* < 0.05) reduced in the 70° turning group. As a result, the hatchability of fertile eggs was significantly higher in the 70° turning group than in the 50° turning group. The relative embryo weight on E22 in the 70° group increased by 2.4% (*p* < 0.01), and the relative yolk weight showed no significant difference between the 70° and 50° turning groups (*p* = 0.06). However, the differences in the relative yolk weight on H0 (*p* < 0.05) and the hatched gosling weight between the 70° and 50° turning groups (*p* < 0.01) were significant.

### 3.2. Validation and Multivariate Analysis of LC-MS Results

**Figure 1:**
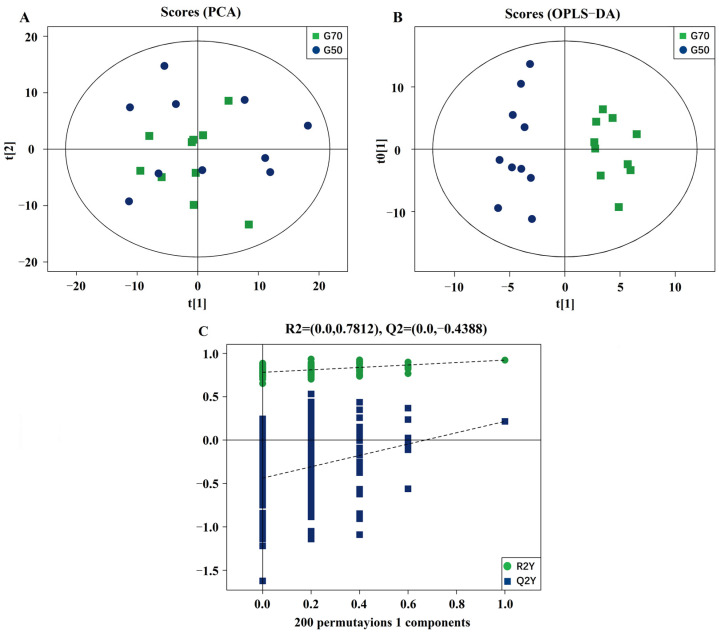
Data modeling and selection of differentially expressed lipid metabolites of yolk on E22 from eggs at a 50° or 70° turning angle. (**A**) Principal component analysis (PCA) of the normalized peak areas of individual metabolites. (**B**) OPLS-DA differentiation of the lipid metabolites of yolk from eggs at a 50° or 70° turning angle. (**C**) Validation of OPLS-DA results based on 200 permutation tests. OPLS-DA, orthogonal projections to latent structures-discriminate analysis. PCA and OPLS-DA analysis were measured with SIMCA14.1 software V14.1 (Sartorius Stedim Data Analytics AB, Umea, Sweden).

PCA plots (Figure 1A) were obtained with two principal components in positive and negative ionization modes and supported the observation (R2X [cum] = 0.578) that the lipid species profile of the yolks separated better in the 70° turning group than in the 50° turning group. In the OPLS-DA analysis, the classification parameters obtained from the software were R2Y (cum) = 0.228 and Q2Y (cum) = 0.948, which were stable and provided good fitness and prediction (Figure 1B). Furthermore, a permutation test was used to validate the model, and the results indicated the robustness of the models and a low risk of overfitting (Figure 1C).

### 3.3. Yolk Lipid Mobilization

**Figure 2:**
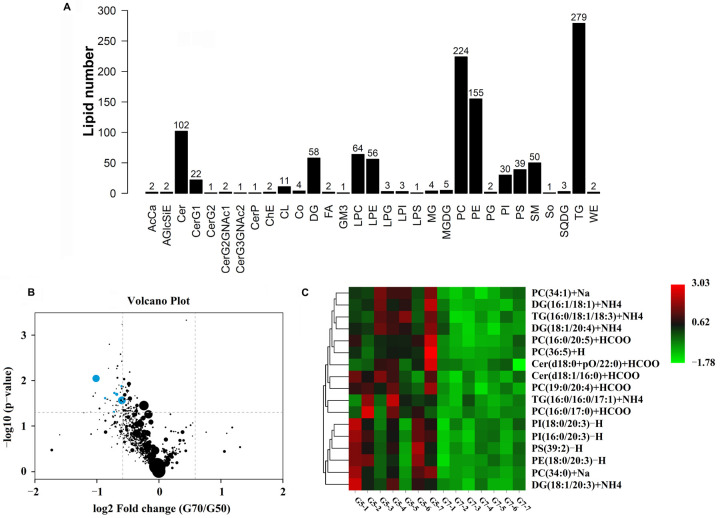
Lipid metabolomics of yolk from eggs in the 50° and 70° turning angle groups. (**A**) Numbers and species of lipid metabolites of yolk. (**B**) A volcano plot of the differences in lipid metabolite abundance between the yolk from eggs in the 50° and 70° turning angle groups. (**C**) Heat map and hierarchical clustering of differentially expressed metabolites in the 50° and 70° turning angle groups. Red, upregulation; blue, downregulation. Hierarchical clustering analyses were performed on significantly expressed metabolites, using Euclidean correlation as the distance measure, based on the Ward clustering algorithm.

**Figure 3:**
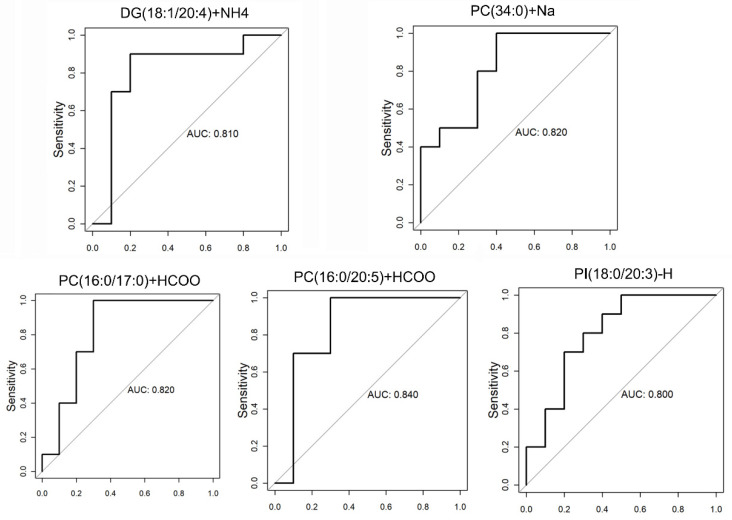
Receiver operating characteristic (ROC) curves for the five significantly expressed metabolites, with an area under the ROC curve of equal to or greater than 80%.

UHPLC-MS identified 1132 lipid species in 31 lipid classes. A total of 17 lipid species differed significantly between the 70° and 50° turning groups (*VIP* > 1 and *p* < 0.05) (Figure 2A). They belonged to the following seven lipid classes: phosphatidylcholine (PC), diglycerides (DGs), triglycerides (TGs), ceramides, phosphatidylinositol (PI), phosphatidylserine, and phosphatidylethanolamine. A volcano map of the difference analyses was plotted to indicate the differences in lipid metabolites in yolk samples between the 50° and 70° turning groups (Figure 2B). A heatmap for the two groups was developed based on normalized data using auto-scale features for standardization (Figure 2C). All differentially expressed lipid species were detected at lower concentrations in yolk samples from the eggs in the 70° turning group than from those in the 50° turning group. More importantly, five differentially expressed lipids (DG [38:5], PC [34:0], PC [33:0], PC [36:5], and PI [38:3] were observed, with scores for the area under the receiver operating characteristic curve that were ≥80% (Figure 3).

### 3.4. Lipid Utilization Genes

**Figure 4:**
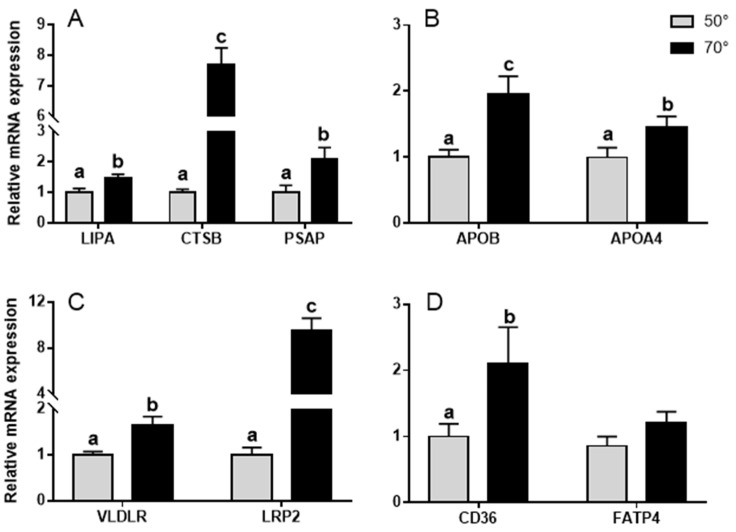
mRNA levels of genes relative to β-actin, (**A**) LIPA, CTSB, and PSAP, (**B**) APOB and APOA4, (**C**) VLDLR and LRP2, and (**D**) CD36 and FATP4 of goose embryo yolk sac membranes between the 50° and 70° turning angle groups. Data are shown as mean values ± standard error of the mean. Different letters above the bars denote significant differences between the different treatment groups (a,b: *p* < 0.05; a,c: *p* < 0.01).

The expression of nine genes involved in lipid metabolism and transportation in the YSM was studied. The expression of several lysosomal digestion-related genes was found to be altered in the YSM at different turning angles. The expression of prosaposin (PSAP), which facilitates the catabolism of glycosphingolipids in lysosomes, increased in the YSM as the turning angle changed to 70° (*p* < 0.05) (Figure 4A). We also found a higher expression of lipase A (LIPA), which catalyzes the hydrolysis of cholesteryl esters and TGs in lysosomes, in the YSM from eggs in the 70° turning angle group (*p* < 0.05) (Figure 4A). In addition, the expression levels of lysosomal proteases, such as cathepsin B (CTSB), were significantly higher (*p* < 0.01) in the 70° turning angle group than in the 50° turning group (Figure 4A).

We also observed that the expression of apolipoprotein genes—apolipoprotein B (APOB) as well as apolipoprotein A4 (APOA4)—was significantly upregulated as the turning angle changed to 70° (Figure 4B). A relatively higher expression level of the very low-density lipoprotein receptor (VLDLR) (*p* < 0.05) in the YSM was observed in the 70° turning group compared to the 50° turning group on E22 (Figure 4C). In addition, the expression level of LDL receptor-related protein 2 (LRP2), a receptor for a range of lipoproteins, was increased in the 70° turning angle group compared with that in the 50° turning angle group (*p* < 0.01) (Figure 4C). In addition, the expression of the thrombospondin receptor (CD36), a membrane fatty acid transporter, was significantly increased (*p* < 0.05) in the YSM from eggs in the 70° turning angle group (Figure 4D).

## 4. Discussion

In this study, we applied a lipid metabolomics approach to elucidate lipid metabolomic changes during incubation using a wider egg turning angle. A total of 1132 lipid species in 31 lipid classes were identified through a UHPLC-MS analysis, including 17 lipid species that exhibited significant concentration variations, which were lower in the yolk of the eggs that were incubated at a 70° egg turning angle than in that of eggs incubated at a 50° egg turning angle. These were associated with the upregulation of the expression levels of eight genes involved in lipid metabolism and transport. Therefore, the incubation of goose eggs at a wider 70° egg turning angle promoted better utilization of yolk lipids that could contribute to enhanced embryonic development with higher hatchability, hatched gosling weight, and quality.

Egg turning can affect the success of incubation and the quality of fowl eggs [25,26]. Embryos are mainly poikilothermic [27], which means that their metabolic rate, yolk utilization, and embryonic growth during incubation are temperature dependent [28]. It was reported that the hatchability of chicken eggs increased progressively with turning angles ranging from 20° to 45° [29,30], and the increase was linear when the turning angles were between 30° and 45° around the short axis of the egg [31]. It was found that albumen utilization was nearly complete under a wider turning angle—for example, 45°—which is much higher than that under a smaller turning angle of 30°, which often leaves an ample amount of unused albumen [31]. Based on these previous findings, we hypothesized that it is necessary to use much wider egg turning angles for the optimal utilization of egg materials, which would facilitate embryonic development in the incubation of much larger or heavier goose eggs. In our previous studies, we observed that wider angle turning during incubation significantly increased embryonic development, hatched gosling weight, and hatchability [12,13]. Enhanced embryonic development or growth was associated with the upregulation of the somatotropic axis hormone genes and muscular development regulation genes [13]. However, nutrient materials for embryonic development and growth must be derived from the albumen and yolk lipids that are pre-stored in the egg. The utilization efficiency of these nutrients in eggs incubated at wider turning angles must be higher than that in eggs incubated at narrower turning angles.

Egg yolk is a rich source of structural and energy-rich lipids, with especially high levels of PC and PE [32]. Egg yolk lipids are the primary source of energy during the second half of the incubation period and the early post-hatching period, and more than 90% of the total energy produced by the embryo and hatchling originates from the oxidation of egg yolk lipids [33,34,35]. Yolk utilization is related to the metabolic and developmental rates of the embryo [36], and a higher metabolic rate leads to a lower residual yolk weight [19,37]. In this study, the relative embryo mass significantly increased by 2.40%, whereas the relative yolk mass decreased by 1.92% on E22 in the 70° turning group compared with those in the 50° turning group (*p* = 0.06). Taking into account the weight of a goose egg, this is an approximately 0.6–0.7 g difference. The lipid metabolite data indicated that the residual yolk lipids in the 70° turning group were lower than those in the 50° turning group, particularly PC (34:0), PC (33:0), and PC (36:5) lipids. These results indicated that wider angle egg turning improved the metabolic rate and promoted yolk utilization in the incubator, especially the absorption and utilization of lipids in the yolk. The higher metabolic rate causes the embryo to produce more heat, which causes the egg temperature to rise in the 70° turning group. Further, the higher egg temperature increased the water loss of the eggs, resulting in a 0.6–0.7 g difference in egg weight between the two groups. Further, the phospholipids that were also mobilized from the yolk were used for the synthesis of the cell membrane materials. The 70° turning embryos had larger body mass and muscle cell diameter [13], which indicated a higher requirement for and utilization of yolk phospholipids, as was the case in this study.

The utilization of yolk lipids is achieved through lipoproteins. Embryonic lipoproteins are synthesized in part in the yolk endodermal epithelium [38], and part of the maternal very-low-density lipoproteins (VLDLs) present in the yolk is remodeled in the endodermal epithelial cells [39]. Yolk lipid uptake was slow during the first 2 weeks of incubation and very rapid during the third and final weeks of incubation. This uptake is facilitated by the endocytosis of low-density lipoproteins (LDLs) through the inner endodermal columnar cells of the YSM to the surrounding blood vessels and embryonic circulation. In hatching chicken, serum concentrations of high-density lipoproteins and LDLs decrease several folds in chicks up to day 5 post-hatching [40], which suggests that the lipoproteins are responsible for much of the lipid transport. Thus, the decrease in lipoprotein concentration suggests that the utilization of embryo circulating lipids is reduced [41]. In the present study, the 70° turning angle increased not only the expression of genes related to lipolysis and fat digestion enzymes in the YSM on E22, such as LIPA, CTSB, and PSAP genes, but also the expression of genes related to lipid transport, such as APOB, APOA4, VLDLR, and LRP2. In addition, we found that the expression of CD36 and FATP4 in the YSM was increased more in the 70° turning angle group than in the 50° turning angle group. These lipolytic enzymes, generated by the yolk sac, function to break down the yolk contents first, and the resultant products are absorbed, hydrolyzed, and formed into VLDL particles that can be released into the embryonic circulation via the extensive network of blood vessels [14,33,42]. CD36 is the predominant protein that facilitates fatty acid uptake. The upregulation of CD36 expression in the 70° turning angle group promoted fatty acid uptake [43]. When yolk lipids and lipoproteins used for their transport increased, the use of fatty acids for energy production accelerated in the 70° turning group.

## 5. Conclusions

Wider-angle egg turning during incubation upregulated the expression of genes related to lipolysis and fat digestion enzymes, such as LIPA, CTSB, and PSAP genes, as well as genes related to lipid transportation, such as APOB, APOA4, VLDLR, LRP2, and CD36. The upregulation of these genes facilitates the efficient utilization of lipids that are stored in the yolk. Higher efficiency of lipid mobilization promotes embryonic development, which leads to higher hatchability rates and improved gosling quality.

## Figures and Tables

**Table 1:** Primer sequences for RT-PCR.

Gene name	Accession Number	Primer Sequence (5′ to 3′)	PCR Product (bp)
β-actin	M26111	TGACGCAGATCATGTTTGAGA GCAGAGCGTAGCCCTCATAG	159
LIPA	XM_013171545.1	CTGAGCTGCTTCTCAAGGACA CGCAGGGCAATGTGTTGAAT	200
CTSB	XM_013184419.1	TGATGTACAAGTCTGGGGTGT CACGATTTCGGACTCGATGC	200
PSAP	XM_027460545.2	CAGGAAGCCGTCAGGACAAA ACAGCCTGATCCTTCATGTGC	167
APOB	XM_013194833.1	AAGCAACAAGGAAGCTCCTGA ATGGCCTGTGCAATCATTTCG	156
APOA4	XM_021270123.3	CAAGCAGATCAACACCCTGC CCTTGCGGATCTGCTCCTT	149
VLDLR	XM_013198844.1	GGCAGTGCAATGGTGTGAGAGAC GGGGCTCATCACTCCAGTCCTTG	171
LRP2	XM_013171457.1	GACGACTGCAAAATGTGGGG TGACGCAATACCAGCCGAAG	138
CD36	XM_013180470	GGGGAAGTCTGGCAACAAAC TCTTCCTGAGTGAAGCTGCTTTG	100
FATP4	XM_013198644.1	TGCACTTTCTGGTGCAAAGC ACATGCGGAAGTACCTGCAAT	170

**Table 2:** Hatching performance, relative yolk, and embryo weight at 70°and 50° turning angles.

Incubation or Hatch Days	Items	Group	
		50°	70°
E7	Fertilization rate (%)	93.42 ± 0.27	91.91 ± 1.46
	Early mortality (%)	3.89 ± 0.25	3.46 ± 0.63
E18	Middle mortality (%)	1.19 ± 0.18	1.44 ± 0.16
E22	Relative embryo weight (%)	27.54 ± 0.54 ^a^	29.94 ± 0.59 ^c^
	Relative yolk weight (%)	27.30 ± 0.65	25.38 ± 0.74
H0	Late mortality (%)	5.66 ± 0.55 ^a^	3.39 ± 0.29 ^b^
	Relative yolk weight (%)	12.77 ± 1.64 ^a^	8.95 ± 0.87 ^b^
	Hatched gosling weight (g)	100.99 ± 1.43 ^a^	106.21 ± 1.66 ^b^
	Hatchability of fertile eggs (%)	89.59 ± 0.52 ^a^	91.92 ± 0.77 ^b^

Note: Values with different superscript letters in each row are significantly different (^a,b^: *p* < 0.05, ^a,c^: *p* < 0.01).

## Data Availability

All data generated or analyzed during this study are included in this published paper.
